# A hazard-based approach to modelling the effects of online shopping on intershopping duration

**DOI:** 10.1007/s11116-017-9838-3

**Published:** 2017-11-21

**Authors:** Esra Suel, Nicolò Daina, John W. Polak

**Affiliations:** Centre for Transport Studies, Imperial College London, Exhibition Road, London, SW7 2AZ UK

**Keywords:** Online shopping, Intershopping duration, Trip frequency, Hazard-based duration models, Travel demand modelling, Consumer panel data

## Abstract

Despite growing prevalence of online shopping, its impacts on mobility are poorly understood. This partially results from the lack of sufficiently detailed data. In this paper we address this gap using consumer panel data, a new dataset for this context. We analyse one year long longitudinal grocery shopping purchase data from London shoppers to investigate the effects of online shopping on overall shopping activity patterns and personal trips. We characterise the temporal structure of shopping demand by means of the duration between shopping episodes using hazard-based duration models. These models have been used to study inter-shopping spells for traditional shopping in the literature, however effects of online shopping were not considered. Here, we differentiate between shopping events and shopping trips. The former refers to all types of shopping activity including both online and in-store, while the latter is restricted to physical shopping trips. Separate models were estimated for each and results suggest potential substitution effects between online and in-store in the context of grocery shopping. We find that having shopped online since the last shopping trip significantly reduces the likelihood of a physical shopping trip. We do not observe the same effect for inter-event durations. Hence, shopping online does not have a significant effect on overall shopping activity frequency, yet affects shopping trip rates. This is a key finding and suggests potential substitution between online shopping and physical trips to the store. Additional insights on which factors, including basket size and demographics, affect inter-shopping durations are also drawn.

## Introduction

Information and communication technologies (ICT) are transforming the retail sector with a proliferation of new channels and virtual alternatives. This change is observed in spending patterns: online retailing in the UK accounted for 14% in 2014 of all sales in 2014 up from 2.1% in 2002, Office for National Statistics 2015, and in the USA share of online retailing reached 7% of all sales up from only 2.8% in 2006 (Bucchioni et al. 2015). The growing prevalence of online shopping is driving major disruption in retail with poorly understood impacts on personal mobility and delivery operations. Historically, it has been difficult to study such impacts due to lack of sufficiently detailed data. In this paper, we present the use of consumer panel data from Kantar Worldpanel (KWP), which is a relatively new data source in this context. Consumer panels are attractive as they capture individual shopping episodes via both online and in-store channels. Further, market research companies that collect consumer panel data (e.g., Kantar, Nielsen, GfK, Ipsos) operate in multiple international markets, and hence this type of data is available for many countries and similar analyses can be repeated in different contexts and times. The focus of this paper is on modelling inter-shopping durations for grocery shopping using consumer panel data incorporating both online and in-store activities. The overall aim is to gain insights into how shopping patterns are changing with the increasing use of online channels for shopping. This will provide insights to substitution question between physical and virtual activity in the context of grocery shopping, and also contribute to multiday activity generation modelling work.

The remainder of this paper is organised as follows. In the next sub-sections, we briefly review earlier studies on ICT’s impact on shopping related personal travel and present specific objectives and rationale behind using hazard-based models as well as our focus on grocery shopping. In the following sections, we lay out the methodology describing analysis methods and empirical data used, followed by presentation of the results and conclusions.

### Previous work

At a conceptual level, transport researchers classified potential implications of ICT on personal travel into four types: substitution, complementarity, modification, and neutrality (Salomon 1985, 1986; Mokhtarian 2004; Hjorthol 2002; Mokhtarian 2009). Building on this categorisation, many researchers have attempted to quantify the net effects of increasing use of ICT on journey frequency and miles travelled. Bhat et al. (2003); Rotem-Mindali and Weltevreden (2013); Rotem-Mindali (2014) provided detailed reviews of such studies that focus more specifically on online shopping. Findings from this line of work, however, are diverse and sometimes contradictory. Results are heavily influenced by how net effects are being measured, what is being categorised as shopping (e.g., searching, transactions, returns), types of products being considered (e.g., furniture, electronics, books, groceries), sample selection, and other assumptions. Another related strand of transport literature investigates the relationship between frequencies of online shopping and traditional in-store shopping to gain further insights to the substitution or complementarity question (Cao 2012; Farag et al. 2007; Circella and Mokhtarian 2010; Zhou and Wang 2014). These studies also aim to explore how certain characteristics of individuals (e.g., socio-demographics, shopping attitudes, internet experience, geographical accessibility) will influence shopping frequencies via online and in-store channels using descriptive analyses, regression methods, and structural equations models. Most studies identify a positive relationship between online and in-store shopping frequency. For instance, Circella and Mokhtarian (2010) reported complementarity and found limited evidence for substitution between online and in-store shopping frequency for experience (clothing/shoes) and search (books/DVDs) goods. Farag (2006) found evidence of frequent in-store shoppers also shopping online more frequently, yet note that the causality is not easy to establish. More recently, Suel et al. (2015) reported evidence of substitution effects in the context of grocery shopping in London.

A common challenge is finding suitable datasets as national and regional travel surveys often capture very limited information regarding online activity and are subject to substantial local variation and idiosyncrasy. Circella and Mokhtarian (2010) and Cao (2012), for instance, relied on one-off ad-hoc survey data where respondents are asked to report how often they are engaged in online and in-store shopping separately on a frequency scale (e.g., ranging from ‘never’ to ‘more than once a week’). In the dataset used by Farag et al. (2007), online shopping frequency is captured using a points scale while information on in-store shopping is derived from a detailed two-day travel diary. Becky and Wang (2017) collected data on time spent for online shopping at home on a scale ranging from never to more than one hour per day. Zhou and Wang (2014) made use of the US National Household Travel Survey (NHTS) in this context, which benefits from a large sample size and detailed travel diary data for in-store shopping, yet only captures online shopping on a points scale. In short, none of the datasets used in cited studies contain information on actual online shopping activity, rather they make use of survey responses that measure online shopping activity levels on a points scale. Notwithstanding the limitations, these studies offered important insights to the complex relationships between online and in-store shopping. Importantly, they all highlighted the difficulty in finding a simple answer to the complementarity or substitution question, the value of recognizing the complexity of the underlying behaviour, and the need for tools to better understand changes triggered by wider use of technology.

### Aim and specific objectives

Against this background, the overall aim of the present study is to investigate the effects of online shopping on the temporal patterns of shopping activity and related travel demand by making use of hazard models. Hazard based models have been previously applied to analyse inter-shopping duration for traditional in-store shopping trips (Kim and Park 1997; Leszczyc et al. 2000; Schonfelder and Axhausen 2001; Bhat et al. 2004b, 2005). In transport research, these studies were motivated by the need to develop multi-day (as opposed to single-day) activity generation model systems to account for weekly shopping patterns (Bhat et al. 2004a). The base line hazard function is used to model the increasing likelihood of participation to shopping with increase in elapsed time since the last shopping episode due to inventory depletion effects. However, to the best of our knowledge, previous work on inter-shopping duration modelling have not considered in-home shopping activity via online channels. Yet, one would expect participation to online shopping to also influence the likelihood of participation to in-store shopping and vice versa. Online shopping might also alter overall shopping patterns, leading to observed shifts in frequencies. One relevant study by Bhat et al. (2003) used hazard based models to analyse the effects of using mobile phones and internet on inter-shopping duration for non-maintenance shopping (excluding grocery and health related shopping etc.). Findings suggested potential substitution between mobile phone use and non-maintenance shopping travel. ICT-use data, however, was at the individual level relating to whether someone uses mobile phone and internet at all. Hence the effects of specific episodes of ICT use were not accounted for. Hazard-based models of inter-shopping duration can be extended to incorporate internet shopping where observed online shopping episodes influence the baseline hazard or modelled as end of duration events.

Shopping itself is a heterogeneous class of activities (Mokhtarian 2004; Visser and Lanzendorf 2004; Rotem-Mindali and Salomon 2007; Girard et al. 2003). For instance, individual behaviours differ substantially when shopping for daily groceries as compared to white goods purchases. Changes in retail supply, therefore, will also likely alter shopping behaviour in different ways in different sectors (e.g., electronics, clothing, furniture). While it is of interest in principle to investigate these differences, a comprehensive treatment is beyond the scope of a single paper. We limit our focus to grocery shopping occasions where a transaction occurs (i.e., excluding pre- and post-purchase trips). The reasons for this decision is as follows. First, it is the most common and frequent type of shopping, hence more relevant for travel implications^1^ (Golob 2002). Second, online grocery market is a fast growing segment at present and is expected to grow with an annual growth rate of more than 50% over the next five years in the UK (Gladding 2016). Hence, potential implications on personal travel and logistics operations will be increasingly important. Third, since grocery shopping has minimal recreational value it allows to really get at the physical vs. virtual issue net of any of the complicating factors relating to recreational value of shopping (Gould and Golob 1997). Fourth, pre-purchase (searching) and post-purchase (returns) stages are less relevant for additional trip generation reducing the complexity involved in modelling and data collection. Fifth, logistics operations for grocery shopping deliveries are different in nature and often more cumbersome (e.g., tighter delivery windows, constraint for in-person deliveries, fresh products). Therefore, policy implications of online grocery shopping associated with extra freight vehicle trips will not only be important but also different when compared to other types of products.

More specifically, the objectives in the present are as follows. First is to incorporate online shopping activity in hazard-based inter-shopping duration models for grocery shopping. This requires a data source where individual shopping events across both online and in-store channels are captured. Second objective is to analyse effects of online shopping separately on (i) inter-shopping duration for all types of shopping events (including both online and in-store activity) and (ii) duration between in-store shopping trips (i.e., out-of-home shopping activity) generating personal travel to stores.

Modelling the duration between consecutive shopping episodes including both online and in-store activity will contribute to multi-day activity generation work for estimating travel demand. It will also help explore the effects of online shopping on frequency and trips generated, potentially providing new insights to the complementarity or substitution question. It will also contribute to the development of tools that enable predicting when the next shopping event or trip will take place. Such prediction capability is crucial for retail delivery operations especially in certain market segments such as online groceries given their struggle to make businesses profitable (Twentyman 2015).

## Methodology

### Data

The UK has a comparatively developed online market for groceries (Kantar Worldpanel 2015), hence offers an opportunity to study impacts of ICT use on personal travel patterns for the purposes of the present study. National and regional travel surveys (e.g., British National Travel Survey (DfT 2016), London Travel Demand Survey (TfL 2016)) often used in transport research offer detailed data on physical trips for food shopping, yet capture very limited information regarding online activity. Specific episodes are not recorded and information regarding when each online shopping event took place, crucial for our purposes here, is not available.

Data used for the present study were obtained from a consumer panel run by Kantar Worldpanel (KWP). Participating households use hand-held optical scanners to record daily purchases of fast moving consumer goods that are brought home. Fast moving consumer goods include products found in supermarkets typically bought frequently and at relatively low prices. Examples are groceries, toiletries, health and beauty items. Panellists are also asked to scan and send till receipts whenever they make a shopping trip or get deliveries. Shopping occasions are recorded regardless of the retail chain, hence include visits to smaller local shops, independent corner shops, and larger supermarket chains. KWP is a continuous panel where households can participate for as long as they wish providing longitudinal data and participants receive incentives in the form of redeemable points. Household characteristics including socio-demographics are collected at the initial interview and continuously updated every six-months or annually. All household members record their purchases separately, however the dataset employed had an indicator for main shopper only but otherwise did not distinguish between other household members. KWP defines main shoppers as household members that are responsible for the bulk of grocery shopping in their household. For model estimations, we use shopping occasions recorded by all shoppers in the household without distinguishing between different members of the household. Purchases from all retailers including online retailers are recorded together with basket sizes in terms of monetary value. Unlike travel surveys, both online and in-store shopping observations contain data at the same level of detail. Shopping records include visits where a transaction occurs since product level purchases are recorded, hence will exclude search only or returns only visits. Since the focus is on grocery shopping, however, shopping occasions which will not involve any purchases are likely minimal. Information on basket sizes is also crucial as the amount of shopping will also potentially influence the duration until the next shopping occasion. For assessing the influence of online shopping on inter-shopping duration, it is important to separately account for basket size effects especially since online shopping is often associated with large basket sizes due to minimum size requirements for delivery. Consumer panel data is also attractive as market research companies operate consumer panels in many countries and some make it available to researchers.^2^ Availability is important for generalisability of our proposed method. Consumer panels, however, were also found to have some shortcomings. Information on the retail chain is available, but the specific store is not known. They also do not gather travel related information, for instance, travel mode choice for shopping is not collected that is potentially important when analysing travel implications. Market research companies collect relatively limited demographics information on respondents and transitions (e.g., changes in employment, income, marital status, children) are not always well recorded.

The sample obtained from KWP covers a one year period between September 2013 and August 2014 from a sample of 168 households in London. 124 households in our analysis sample are panellists who reside in the two selected boroughs, Barnet and Enfield. Thirty four out of 124 were online shopping households with reported online observations during the one year period. An additional forty four households were drawn randomly from London among online shopping households since we are primarily interested in their behaviour. This, however, causes sampling bias as online shopping households are over represented in our estimation sample. To correct for this, we use weights for all observations assuming that the share of online shopping households in London matches observed shares in Enfield and Barnet.^3^ When we compare demographics for our sample and Census data from Enfield and Barnet in 2011 (Office for National Statistics 2011), we found that households with older main shoppers and highest social classes are overrepresented in our study sample (Suel 2016). We note that no information is available from the Census on main shoppers, hence age of household reference person is used as proxy for comparison.

While non-traditional panel data offers significant advantages for research, potential problems and sampling biases should not be overlooked. We note that our sample will share important characteristics relating to the geographical proximity of residential addresses. Also, while the number of recurring observations from each responding household was large, the main limitation is that the number of households in the sample was relatively small due to budget constraints. If population scale prediction is a prioriry, then a larger and more representative sample would be desirable. Here, we demonstrate the insights drawn and prediction capabilities that can be developed with existing data sources using modelling tools as presented below.

### Analysis methods

Hazard-based methods have been initially developed for modelling duration data such as time to failure or time to some form of state change. These methods are often used in survival analysis in the context of biological problems (e.g., expected duration of time to death or organ failure) and reliability analysis for mechanical systems. Hensher and Mannering (1994); Bhat and Pinjari (2000) presented extensive reviews of hazard-based duration models and their applications in transport research. The basic idea is to model the probability of an end-of-duration occurrence given that the duration lasted for some time. This probability will depend on the length of elapsed time and also on relevant co-variates. These models are suitable for analysing inter-shopping duration, i.e. time between two consecutive shopping occasions. The probability of ending a duration since last shopping activity is dependent on length of the duration (time elapsed since last activity) due to depletion effects (i.e. an individual is more likely to go grocery shopping on any given day if s/he has not done so for a longer period). This duration will also likely depend on other observable characteristics such as household size (larger households consume more and might need to go shopping more frequently) and basket size on last activity (if more products are stocked the need to go shopping again might decrease).

In this paper, we aim to model gap times between recurring grocery shopping occasions using the Cox proportional hazards model. The probability of an end-of-duration occurrence (i.e., a shopping observation) will take place at time *t* given that it has lasted until *t* is described by the hazard function *h*(*t*). Estimating effect sizes associated with selected determinants of duration such as demographic variables and situational factors are of interest for studying inter-shopping duration behaviour. In the Cox proportional hazards model, it is assumed that covariates act multiplicatively on an underlying hazard function, which can be represented as in Eq. (1). Note that using the Cox approach, the hazard distribution itself need not to be estimated for estimating the effects of different factors for an end-of-duration occurrence (Cox 1972).

1 $$\begin{aligned} h(t|Z)= h_{o}(t) \exp (\beta Z) \end{aligned}$$

where $h_{o}(t)$: baseline hazard; Z: vector of covariates; $\beta$: parameters to be estimated.

The proportional hazard form presented in Eq. (1) is based on the assumption that gap times are independent and identically distributed. This might be restrictive, for instance, if estimation data contains multiple observations of recurring shopping occasions from the same shopper as is the case here. Correlation between observations belonging to the same shopper due to unobserved factors can be accounted for using so called frailty models. For this case, we use the form in Eq. (2) for the proportional hazard function (Cook and Lawless 2007).

2 $$\begin{aligned} h(t|Z)= h_{o}(t) \exp (\beta Z + b_i) \end{aligned}$$

where $b_i$ is the unobserved random effect associated with individual *i* and are assumed to follow a Gaussian distribution. Additionally, random effect terms capture the influence of unobserved risk factors i.e. omitted variables affecting the hazard. While only few risk factors can be observed and meaningfully measured, other unknown or unmeasured variables may influence the time between shopping occasions. Modelling individual unobserved random effects does not inform on specific omitted variables (on how many, which and how important), but highlights that some significant factors have been excluded (Hougaard 1995); it will however reduce omitted variables’ bias by capturing effects of omitted variables independent from the observed covariates. We report estimation results using both forms in Eq. (1) and Eq. (2) when discussing our findings.

**Fig. 1:**
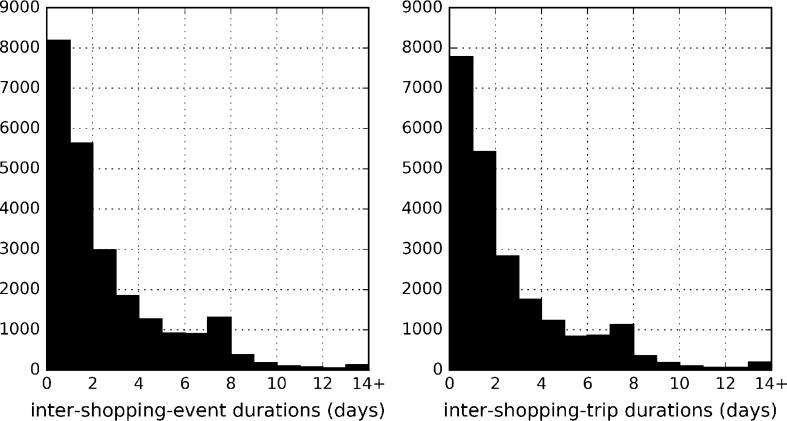
Histogram plots for inter-shopping-event and inter-shopping-trip durations

For our empirical analyses, we differentiate between *shopping events* and *shopping trips*. For the former, gap times are defined as time between two shopping events regardless of whether they were online or in-store. Online and in-store observations are both modelled as end-of-duration occurrences. For the latter, on the other hand, gap times are defined as time elapsed between two in-store shopping trips. Only in-store observations, which generate personal trips to physical stores, are modelled as end-of-duration occurrences. From available data, we calculated times between shopping events and shopping trips separately over one year. These are also used as durations for hazard based analyses. Figure 1 shows the distribution of inter-shopping-event and inter-shopping-trip times over our sample. As expected, times between shopping trips are longer in duration on average and there are cases where households do only online shopping for long periods without physically visiting stores. Note that there were also more outlier cases for inter-shipping-trip durations grouped in 14+ days bin with more cases where time between consecutive shopping trip exceed two weeks. When multiple shopping events or trips are observed in a single day, durations are computed as being evenly distributed across one day. The first bins in histogram plots include shopping activity observed within one day, which makes up almost 35% of all observations for each category.

Our main interest in this paper is to test the effect of online shopping activity on the instantaneous rate of occurrence of shopping (inter-shopping-event durations) and travel for shopping (inter-shopping-trip durations). We do so by incorporating, along with other shopper characteristics and situational variables, two key indicators: (i) whether the household have adopted online shopping as a channel; and (ii) whether their last shopping was online. The former is a dummy variable and is equal to one only after a respondent is observed shopping online and remains equal to one for the remainder of our observation period.^4^ The former variable therefore captures a general long term effect of adoption, whereas the latter would reveal short term (tactical) effects of buying online.

Specification search was conducted using household characteristics and basket size in terms of monetary value. The panel collects information on income, but panellists are not required to report this information. Income information was missing for the sig of the households in the dataset we were provided with. Therefore, social class was used as a proxy for income where higher social classes are assumed to be associated with higher income bands.^5^ We did not have information on gender. The socio-demographic variables used in specification search included: social class, household size, number of children, age, education level, and life stage. Covariates were left out where no significant effects were identified (social class, number of children, education level, and life stage). The final model specification, in addition to two key online channel related indicators, included: household size, age of main shopper, and basket size in the last shopping occasion, all as scale variables. Basket characteristics is often neglected in transport literature, because this type of information is not usually available in traditional datasets used in travel demand analysis. However, as briefly outlined above and presented in the results section basket size variable bears a strong effect. A summary of descriptive statistics for covariates used in the estimation of hazard models is presented in Table 1, along with numbers of shopping events and shopping trips.

**Table 1:** Descriptive statistics for the sample

Number of households		168
Mean age (main shopper)		51.01
Mean household (HH) size		2.50
Online adopters (based on the year before)		66 (39.29%)
Online shoppers (based on the year of analysis)		73 (43.45%)
Number of shopping events		24099
Online shopping events		1140 (4.73%)

	Online shoppers	All shoppers
Average basket size	23.54	22.40
Median basket size	10.75	11.42
Average basket size (online only)	61.02	60.12
Median basket size (online only)	61.30	60.59
Average basket size (in-store trips only)	19.87	20.52
Median basket size (in-store trips only)	9.58	10.75
Average number of shopping events per week	3.46	3.10

## Model results and discussion

We estimated hazard models separately for (i) inter-shopping-event durations and (ii) inter-shopping-trip durations as shown in Tables 2 and 3. Results are presented for fixed effects only proportional hazard and frailty specifications. For both (i) and (ii), incorporating mixed effects using frailty models improve the fits significantly; null hypotheses of the fixed effects only models were rejected using log-likelihood ratio tests. In addition, there were some substantial differences in estimation results as detailed below.

Estimation results for inter-shopping-event durations using the fixed effects only proportional hazard model (Table 2) show increased hazard of shopping events for larger households and older main shoppers; household size has a larger effect. As expected, the larger the basket size in the previous shopping occasion the lower the likelihood of a shopping event. Being an online shopper is positively associated with the likelihood of a shopping event, significant only at the 0.05 level. Having shopped online in the previous observed shopping event, on the other hand, has a negative effect on the underlying hazard and only significant at the 0.05 level. Comparing results with the frailty model, the signs for all parameter estimates are unchanged. However, the significant effects of online related covariates disappeared. In the frailty model, neither adoption nor online shopping in the previous episode has a significant effect on inter-shopping-event durations. Such difference highlights the importance of accounting for unobserved heterogeneity since not doing so might lead to inaccurate interpretations.

**Table 2:** Estimation results for inter-shopping-event durations

	Proportional hazard (fixed effects only)			
	coef	exp(coef)	z-value	*p* value
Household size	0.1295	1.138	3.94	0.0001
Age (main shopper)	0.0102	1.010	3.06	0.0022
Basket size (previous shopping occasion)	− 0.0106	0.989	− 10.87	< 2e−16
Online dummy (previous shopping occasion)	− 0.2117	0.809	− 2.22	0.0265
Online adopter dummy	0.1878	1.207	2.21	0.0273

	Frailty (mixed effects)			
	coef	exp(coef)	z value	*p* value
Household size	0.1550	1.168	3.61	0.0003
Age (main shopper)	0.0136	1.014	3.12	0.0018
Basket size (previous shopping occasion)	− 0.0049	0.995	− 14.24	< 2e−16
Online dummy (previous shopping occasion)	− 0.0410	0.960	− 1.03	0.3100
Online adopter dummy	0.0718	1.074	1.36	0.1700
		95% confidence interval		
Random effects: standard deviation	0.619	0.554	0.686	

**Summary statistics**				
Number of observations	23946			
$\mathcal {L}(0)$	− 153379			

		$\mathcal {L}(\hat{\beta })$	AIC	BIC
Proportional hazard (fixed effects only)		− 152354	304718	304758
Frailty (mixed effects)		− 150455	300923	300971

$^\mathrm{a}$Computed using the profile likelihood method (Therneau 2015)

For inter-shopping-trip duration models (Table 3), household size, age, and basket size variables have similar scales and sizes for both fixed-effects only and frailty models. The larger the household and the older the main shopper the larger is the inter-shopping hazard of a shopping trip. Conversely, the larger the basket size on the previous shopping event the lower is the inter-shopping hazard for a trip. Being an online shopper in the fixed effects only model had a positive and significant effect on the inter-shopping hazard of a shopping trip, the significance is largely reduced in the frailty model. Here, the increased rate of occurrence of shopping trips effectively means that online shoppers engage more frequently in in-store shopping. This finding can be attributable to the unobserved shared tastes of online adopters (e.g., people who enjoy shopping more are the ones who shop more often and also more likely to adopt new shopping channels early). Alternatively, it can be explained by online shopping leading to generation of increased shopping activity (e.g., once people adopt online shopping they start shopping more frequently). Our data and modelling results are not sufficient to discern which case apply, further work is required to explore causality. This effect, however, is only significant at the 0.1 level.

Crucially, having shopped online since the last shopping trip significantly reduces the likelihood of a physical shopping trip. This effect becomes even stronger in the frailty model. We do not observe the same effect for inter-event durations. Hence, shopping online does not have a significant effect on overall shopping activity frequency, yet affects shopping trip rates. This is a key finding and suggests potential substitution between online shopping and physical trips to the store. In line with intuition, when households order their groceries online it delays the next physical trip for in-store shopping. It therefore suggests that online shopping activity might affect trip rates.^6^

**Table 3:** Estimation results for inter-shopping-trip durations

	Proportional hazard (fized effects only)			
	coef	exp(coef)	z-value	*p* value
Household size	0.1300	1.139	3.91	0.0001
Age (main shopper)	0.0099	1.010	2.95	0.0032
Basket size (previous shopping occasion)	− 0.0108	0.989	− 10.43	< 2e−16
Online dummy (previous shopping occasion)	− 0.8463	0.429	− 10.22	< 2e−16
Online adopter dummy	0.2103	1.234	2.47	0.0135

	Frailty (mixed effects)			
	coef	exp(coef)	z value	*p* value
Household size	0.1699	1.185	3.66	0.0003
Age (main shopper)	0.0144	1.015	3.26	0.0011
Basket size (previous shopping occasion)	− 0.0056	0.994	− 15.52	< 2e−16
Online dummy (previous shopping occasion)	− 1.0038	0.366	− 21.65	< 2e−16
Online adopter dummy	0.0938	1.098	1.74	0.0820
		95% confidence interval		
Random effects: standard deviation	0.639	0.566	0.725	

**Summary statistics**				
Number of observations	22815			
$\mathcal {L}(0)$	− 142477			

		$\mathcal {L}(\hat{\beta })$	AIC	BIC
Proportional hazard (fixed effects only)		− 141176	282363	282403
Frailty (mixed effects)		− 139480	278972	279020

$^\mathrm{a}$Computed using the profile likelihood method (Therneau 2015)

## Summary and conclusions

This paper presented the use of consumer panel data and hazard based duration models to explore the potential impacts of online shopping separately on shopping trip frequency and overall shopping activity patterns. Results provide new insights to substitution or complementarity question. Additionally, methods presented can be used for predicting next online order or shopping trip, which is crucial for activity generation models used for travel demand predictions and also for delivery operations. Our study has a number of novel components in comparison with past work that focused on understanding the implications of wider use of ICT on personal travel.

First, while transport researchers have used hazard based duration models to analyse inter-shopping duration in the context of multi-day activity generation, they have not considered online shopping activity and its effects on overall activity generation. Our study accounts for the influence of online activity on temporal patterns of shopping activities.

Second, our analyses are based on a dataset of unprecedented richness in transport research with respect to capturing both online and physical shopping activities. In consumer panels, online shopping activity data are collected in an episode-based manner, rather than through retrospective questionnaires where respondents report how frequently they shop using a points scale. This enables development of more advanced models to help us better understand changes in behaviour triggered by ICT use. Additionally, shopping basket characteristics, likely to affect shopping behaviour yet often neglected in transport literature, are available and used in empirical estimations. Importantly, market research companies operate consumer panels in many countries and make it available to researchers. This is crucial for the generalisability of our proposed method.

Third, we differentiate between shopping events and shopping trips, with the former referring to all types of shopping activity including both online and in-store and the latter restricted to physical shopping trips. Separate models were estimated for each, and results suggest potential substitution effects between online and in-store shopping potentially affecting trip rates. We find that online grocery shopping episodes increase inter-shopping-trip durations and do not significantly influence inter-shopping-event durations. These conclusions are specific to grocery shopping and it is possible that use of ICT has different implications, for instance, on non-maintenance shopping. Future work might therefore focus on obtaining specifically rich datasets enabling similar analyses for other types of shopping.

We finally would like to emphasise that our analyses demonstrated the practical use in transport studies of non transport datasets that are becoming increasingly available. Apart from consumer panels operated internationally (similar to that used here), researchers in marketing and retailing already work with data from online retailers, credit card transactions, and loyalty cards. Desirable collaborations between transport and retail researchers is crucial to unveil the value of existing data sources for transport research.

## Footnotes

1 In Britain, food shopping was the most popular purpose accounting for half of all shopping trips and car was the main mode of transport for 78% of these journeys in 2014 (DfT 2016).

2 For instance, Nielsen’s consumer panel data from the US is available freely to accredited academic researchers through Chicago Booth School of Business (Kilts Center 2017).

3 Non-online shopping and online shopping households have associated weights of one and of 34/(34+44).

4 Note that this adoption variable does not generate endogeneity because the indicator value depends only on past observations.

5 Social Class categories available were based on approximated social grade produced by ONS. AB: Higher and intermediate managerial, administrative, professional occupations; C1: Supervisory, clerical and junior managerial, administrative, professional occupations; C2: Skilled manual occupations; DE: Semi-skilled and unskilled manual occupations, unemployed and lowest grade occupations.

6 We estimated the same four models using our sample with age capped at 65 to better understand effects of our sample over representing older age groups. Coefficient estimates were largely consistent with our full sample findings. The only substantial difference in the size of the coefficient estimates was for the *Online Dummy (previous shopping occasion)* in the inter-shopping-trip durations model, and the effect is much stronger with a larger negative coefficient. This can be explained by older people making more physical trips even after they purchase groceries online potentially due to recreational aspects of it and their time budgets being more flexible. This finding suggests a stronger evidence of substitution for younger households.
